# Polyphenol Microbial Metabolites Exhibit Gut and Blood–Brain Barrier Permeability and Protect Murine Microglia against LPS-Induced Inflammation

**DOI:** 10.3390/metabo9040078

**Published:** 2019-04-19

**Authors:** Shelby L. Johnson, Riley D. Kirk, Nicholas A. DaSilva, Hang Ma, Navindra P. Seeram, Matthew J. Bertin

**Affiliations:** 1 George and Anne Ryan Institute for Neuroscience, University of Rhode Island, Kingston, RI 02881, USA; shelby_johnson@uri.edu (S.L.J.); Hang_ma@uri.edu (H.M.); 2 Department of Biomedical and Pharmaceutical Sciences, College of Pharmacy, University of Rhode Island, Kingston, RI 02881, USA; rileykirk@uri.edu (R.D.K.); nickdasilva91@gmail.com (N.A.D.)

**Keywords:** polyphenol, gut microbial metabolites, permeability, equol, enterodiol, enterolactone, inflammation

## Abstract

Increasing evidence supports the beneficial effects of polyphenol-rich diets, including the traditional Mediterranean diet, for the management of cardiovascular disease, obesity and neurodegenerative diseases. However, a common concern when discussing the protective effects of polyphenol-rich diets against diseases is whether these compounds are present in systemic circulation in their intact/parent forms in order to exert their beneficial effects in vivo. Here, we explore two common classes of dietary polyphenols, namely isoflavones and lignans, and their gut microbial-derived metabolites for gut and blood–brain barrier predicted permeability, as well as protection against neuroinflammatory stimuli in murine BV-2 microglia. Polyphenol microbial metabolites (PMMs) generally showed greater permeability through artificial gut and blood–brain barriers compared to their parent compounds. The parent polyphenols and their corresponding PMMs were evaluated for protective effects against lipopolysaccharide-induced inflammation in BV-2 microglia. The lignan-derived PMMs, equol and enterolactone, exhibited protective effects against nitric oxide production, as well as against pro-inflammatory cytokines (IL-6 and TNF-α) in BV-2 microglia. Therefore, PMMs may contribute, in large part, to the beneficial effects attributed to polyphenol-rich diets, further supporting the important role of gut microbiota in human health and disease prevention.

## 1. Introduction

In the United States, obesity is a growing epidemic. There have been many studies that link obesity to an increased risk of developing other diseases, such as cardiovascular disease, diabetes, and Alzheimer’s disease [1]. A quantitative systematic review estimated that the United States spent $113.9 billion dollars total, or 4.8% of all healthcare spending in 2008 on overweight and obesity care [2]. The standard American diet consisting of high levels of saturated fat, sodium and sugars, contributes to these staggering obesity numbers [3]. People abiding by alternative diets in countries such as Greece and Italy exhibit decreased risks of obesity as well as confounding diseases such as metabolic syndrome [4].

Increasing evidence supports the beneficial effects of a traditional Mediterranean diet in the management of cardiovascular disease, obesity and most recently, neurodegenerative diseases [5]. The Mediterranean diet is characterized by high intake of polyphenols and unsaturated fats, with most of the beneficial effects from this diet being attributed to the high polyphenol intake [6,7]. There has been an increased interest in polyphenols for the treatment of neurodegenerative diseases as they exhibit the ability to cross the blood–brain barrier (BBB) and have a wide range of bioactive properties including antioxidant, anti-inflammatory, anti-apoptotic and lipid-lowering properties [8,9].

Polyphenols are a large class of secondary metabolites produced via the shikimate-derived phenylpropanoid or polyketide pathways, and they are characterized by the presence of two or more benzene rings bearing hydroxyl group(s) and lack any nitrogenous functional group in their core structure [10]. Polyphenols are further divided into several subclasses including stilbenes, flavonoids, lignans, and phenolic acids [11]. Although polyphenols exhibit biological effects in a variety of assays, their poor bioavailability, extensive phase-2 metabolism, and whether they achieve physiologically relevant concentrations as their intact/parent forms to exert their protective effects, have been questioned. Rather, a growing consensus is that dietary polyphenols are metabolized by microbiota in the colon to yield bioactive gut microbial metabolites [12,13,14,15,16]. Herein, two common classes of dietary polyphenols were investigated, isoflavones and lignans, as well as their known polyphenol microbial metabolites (PMMs).

**Figure 1:**
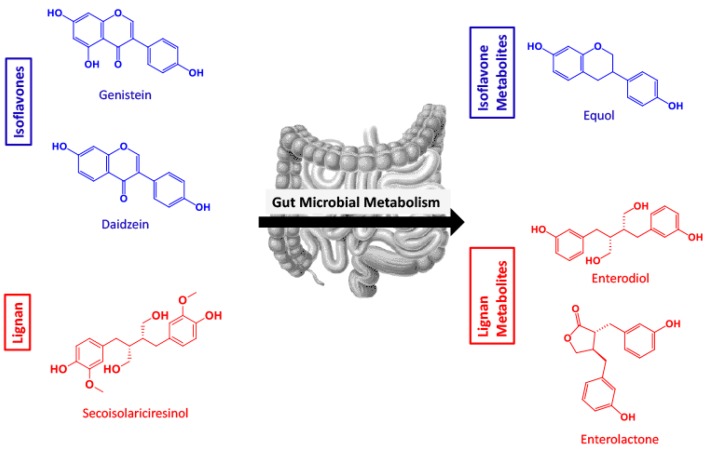
Overview of the polyphenol isoflavones and lignan parent compounds and their respective polyphenol microbial metabolites produced by gut microflora. (Genistein = GEN, daidzein = DAI, equol = EQ, secoisolariciresinol = SECO, enterodiol = ED, enterolactone = EL).

The isoflavones, genistein (GEN) and daidzein (DAI), are two soy-derived polyphenols that have been extensively studied for their bioactivities. Structurally similar to estrogen, isoflavones are comprised of a 3-phenylchromen-4-one backbone, modified by glycosides, O-substituents, and prenylated derivatives [17]. They exhibit the ability to serve as antioxidants, alleviate oxidative stress, and reduce the risk of hormone-dependent cancers [18,19,20]. GEN has been identified as a potential nutraceutical for Alzheimer’s disease as it exhibits the ability to inhibit mitochondria-dependent apoptosis, and alleviate β-amyloid neurotoxicity, providing neuroprotection [21,22,23]. DAI also shows pro-apoptotic and neurotoxic effects against glutamate treatment in mouse hippocampal and cerebral cell cultures [24]. When metabolized in the gut by intestinal microflora, these isoflavones are converted into equol (EQ) (Figure 1) [25]. EQ has exhibited bioactive properties in cardiovascular disease, bone health, and cancers [26]. A recent study identified EQ as protective against oxidative stress in microglia, through the downregulation of neuronal apoptosis, and increased neurite growth [14].

Lignans often have complex structures made up of C6 and C3 units [27]. In plant tissues, lignans are often found as dimers and can be in the free state or sugar bound [28]. Data suggests a lignan-rich diet has numerous benefits such as prevention of hormone-dependent tumors, decrease in plasma cholesterol and glucose profiles, and delaying type 2 diabetes [29,30,31]. The common and prototypical dietary lignan, secoisolariciresinol (SECO), is known to be deglycosylated by bacteria and converted to the PMMs enterodiol (ED) and enterolactone (EL) (Figure 1) [32].

A common question regarding dietary polyphenols is whether they are present in systemic circulation in physiologically relevant concentrations to exert their biological effects. Polyphenol concentrations in the blood generally range from 0.1–1.0 μM [33]. However, certain phenolic metabolites such as pyrogallol sulfate and catechol sulfate reached plasma concentrations ranging from 5–20 μM, while their parent compounds were undetected [34]. Absorption through the GI tract is complex as the pH changes as compounds traverse through the stomach to the intestines, which changes the bioavailability of substances [35]. The high-throughput parallel artificial membrane permeability assay (PAMPA) is used to determine permeability properties related to the transcellular in vivo absorption process of large compound libraries [36]. PAMPA has become a robust, versatile method for predicting passive permeability of compounds through the gastrointestinal (GI) track, the BBB, and skin [37,38]. A major concern for the development of pharmaceuticals for CNS-related diseases is the ability of compounds to cross the BBB, therefore it is of high importance to evaluate this ability early in drug discovery [39]. The BBB is a lining of endothelial cells that protects the brain from the peripheral nervous system [40]. PAMPA uses a combination of phospholipids specific to the membrane being tested along with a microfiber filter to simulate the biological membrane [41].

Herein, we propose that certain PMMs derived from gut microflora metabolism of their parent polyphenols are gut and BBB permeable and may provide protection against neuroinflammatory stress. To explore this hypothesis, we first performed in silico screening for gut and BBB permeability and then PAMPA of the parent polyphenols and their respective PMMs. Neuroprotective activity for all of the compounds was then assessed by evaluating the levels of nitric oxide species and inflammatory cytokines against LPS-induced inflammation in BV-2 murine microglia.

## 2. Results

### 2.1. SwissADME Predicts Polyphenol Microbial Metabolites Are Highly Gut and BBB Permeable

Utilizing SwissADME in silico modeling, BBB and gut permeability were predicted (Table 1). All six of the compounds (parent polyphenols and their corresponding PMMs; Figure 1) were identified as having high gut absorbance. The positive PAMPA gut controls, verapamil, ranitidine, ketoprofen and antipyrine, all exhibited high gut permeability in the SwissADME predictor. ED, DAI and EQ were predicted to have BBB permeability. The PAMPA BBB controls, both BBB permeable and impermeable, were additionally screened in SwissADME, predicting passive permeability for verapamil and corticosterone, but no BBB passive permeability by theophylline. After this predictive measure, compounds were experimentally evaluated for passive permeability in the gut and BBB.

### 2.2. SECO, GEN, DAI, EL, and EQ Exhibit High Permeability through PAMPA Gut

**Figure 2:**
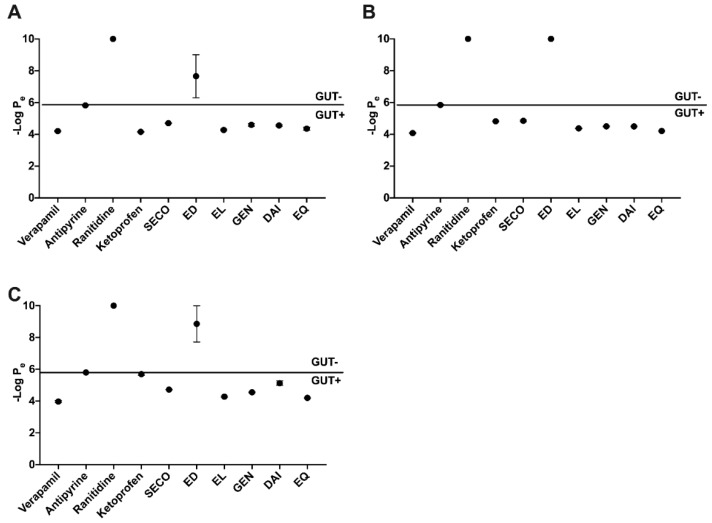
Parallel artificial membrane permeability assay (PAMPA) gut passive permeability at three relevant pH levels: 5.0 (**A**), 6.2 (**B**), and 7.4 (**C**). Controls verapamil, antipyrine and ranitidine exhibit high, medium and low penetrability, respectively. Ketoprofen shows variable permeability with pH change.

Compounds were evaluated for their permeability through simulation membranes of the gut using PAMPA at pHs 5.0, 6.2, and 7.4 (Figure 2). Verapamil was used as the highly permeable control, ranitidine as the low permeability, and antipyrine was used as an intermediate control. Ketoprofen was used as a pH-dependent control. At pH 5, EL showed the highest permeability with a -log P_e_ value of 4.27 ± 0.05, closely followed by EQ at 4.35 ± 0.11. At pH 6.2, EQ showed the highest permeability with a -log P_e_ value of 4.20 ± 0.02 followed by EL at 4.37 ± 0.02. At pH 7.4, the same trend followed as pH 6.2. SECO showed moderate permeability at all pHs tested, and DAI was most permeable at pHs 5 and 6.2. Parent compounds showed high permeability in all three pH treatments, while the metabolite ED showed the lowest permeability of all test compounds in all three pH treatments tested (Figure 2).

### 2.3. GEN, EL, and EQ Show BBB Passive Permeability in PAMPA Assay

**Figure 3:**
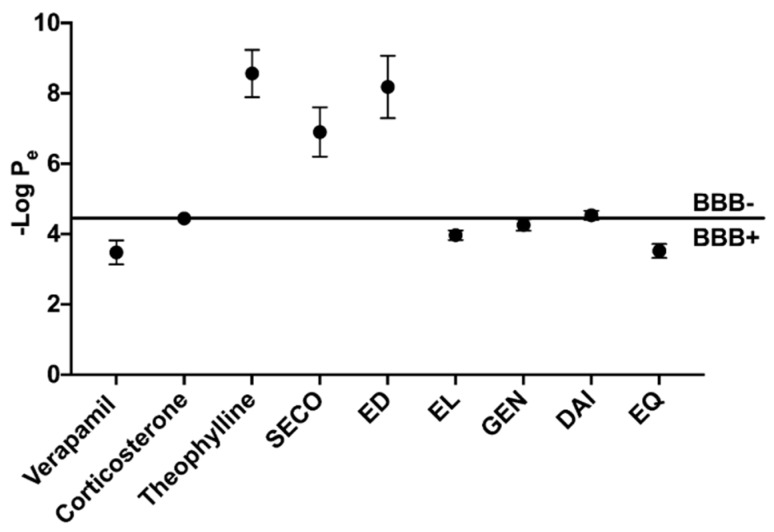
Assessment of blood–brain barrier passive permeability of polyphenols determined by PAMPA. Positive (verapamil), intermediate (corticosterone) and low (theophylline) permeability controls.

Isoflavones and lignans were evaluated for BBB penetration through PAMPA experiments (Figure 3). Verapamil, corticosterone and theophylline were controls for high, intermediate and low permeability, respectively, and yielded similar permeability measures as previously reported [35,39]. EL and EQ exhibited high permeability, with -log P_e_ values of 3.97 ± 0.14 and 3.52 ± 0.20, respectively. Intermediate BBB permeability was seen for both GEN and DAI, with -log P_e_ values of 4.26 ± 0.16 and 4.53 ± 0.13, respectively. SECO and ED exhibited low permeability through the BBB.

### 2.4. Isoflavones and Lignans Show No Cytotoxicity in Murine Microglia

**Figure 4:**
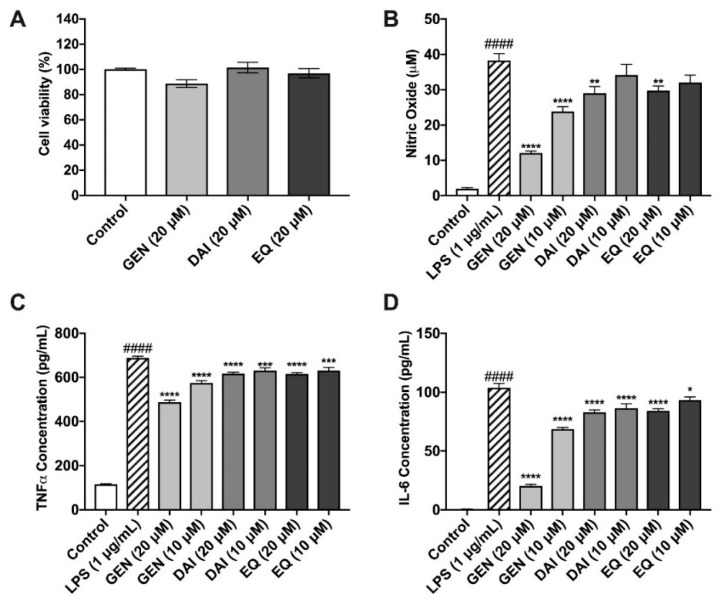
Effects of isoflavones against LPS-induced oxidative stress in BV-2 murine microglia. Isoflavones exhibited no cytotoxic effects in murine microglia (**A**). Isoflavones (20 µM and 10 µM) reduce nitric oxide production, as determined by the Griess Reagent (**B**). Isoflavones inhibited the production of the pro-inflammatory cytokines, TNF-α (**C**) and IL-6 (**D**), in murine microglia. All data expressed as mean ± standard error (n ≥ 3), significance was reported by analysis of variance (ANOVA) followed with Dunnett multiple comparison testing. Significance as compared with control *p* ≤ 0.0001 (####); as compared with LPS, *p* ≤ 0.03 (*), *p* ≤ 0.002 (**), *p* ≤ 0.0002 (***) and *p* ≤ 0.0001 (****).

**Figure 5:**
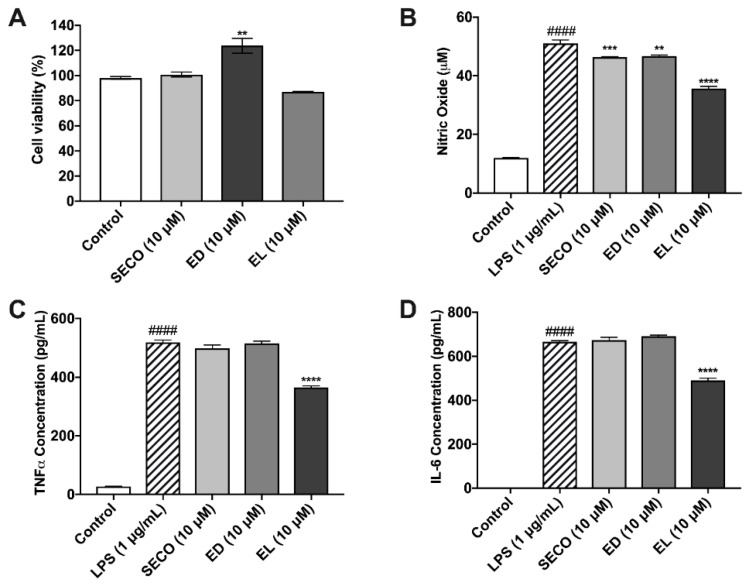
Effects of lignans in murine microglia cells. Lignans were evaluated for cytotoxicity in BV-2 (**A**). Effect of lignan treatment (10 μM) on nitric oxide production, as determined by the Griess Reagent (**B**). Lignans reduce the production of pro-inflammatory cytokines in murine microglia, TNF-α (**C**) and IL-6 (D). All data expressed as mean ± standard error (*n* ≥ 3), significance was reported by analysis of variance (ANOVA) followed with Dunnett multiple comparison testing. Significance as compared with control *p* ≤ 0.0001 (####); as compared with LPS, *p* ≤ 0.002 (**), *p* ≤ 0.0002 (***) and *p* ≤ 0.0001 (****).

Isoflavones were administered at 20 μM, while lignans were dosed at 10 μM. Parent polyphenols and PMMs were evaluated for cytotoxicity in murine microglia after a 24 h incubation period (Figure 4A, Figure 5A). There were no significant reductions in cellular viability at each of the test concentrations, thus indicating nontoxic levels.

### 2.5. Isoflavones Reduce Nitric Oxide Species Production

Isoflavones were evaluated for their ability to reduce nitric oxide production after LPS induction, as determined by the Griess Reagent (Figure 4B). Cells treated with LPS produced 41.5 ± 5.6 μM nitric oxide species, significantly more than the control (0.4 ± 0.02 μM). GEN significantly reduced nitric oxide at both 20 and 10 μM by 68% and 38%, respectively as compared to LPS alone. At 20 μM, DAI and EQ also significantly reduced nitric oxide compared to LPS by 24 and 22%, respectively.

### 2.6. Isoflavones Reduce Pro-Inflammatory Cytokine Release

TNF-α was increased significantly to 687.7 ± 8.0 pg/mL after LPS stimulation, compared to the unstimulated control, 115.4 ± 2.8 pg/mL (Figure 4C). All three isoflavones significantly reduced TNF-α production compared to the LPS-induced treatment. GEN reduced TNF-α, by 29.1% and 16.6% at concentrations of 20 and 10 μM, respectively. DAI and EQ exhibited similar, albeit less potent, protective abilities at both the high (20 μM) and low (10 μM) concentrations (Figure 4C). IL-6 production was significantly increased by LPS (103.7 ± 3.6 pg/mL) compared to the control (0.6 ± 0.05 pg/mL) (Figure 4D). All isoflavones were able to significantly reduce IL-6 production compared to the LPS-induced treatment. GEN was the most effective, reducing IL-6 production by 80.4 and 34.0% at 20 and 10 μM, respectively (Figure 4D).

ANOVA followed by Tukey’s post hoc test was used to determine significant differences amongst test compounds and anti-inflammatory potential. The 20 μM dose of GEN resulted in significantly reduced concentrations of nitric oxide species, TNF-α, and IL-6 compared to all other test compounds and concentrations (Tables S1–S3 in supplementary files), clearly showing GEN as the most potent anti-inflammatory metabolite of the isoflavones and PMMs tested.

### 2.7. Lignans Limit Nitric Oxide Species Production

Nitric oxide concentrations for vehicle-treated microglia were approximately 11.97 μM ± 0.07, whereas LPS stimulation increased this concentration to 51.01 μM ± 1.23. SECO, ED and EL decreased nitric oxide release by approximately 9.13 (46.35 μM ± 0.22), 8.54 (46.65 μM ± 0.36) and 30.07% (35.67 μM ± 0.74), respectively (Figure 5B).

### 2.8. SECO, ED and EL Significantly Reduce IL-6 and TNF-α

As expected, IL-6 and TNF-α concentrations were elevated in LPS-treated microglia with concentrations of 665.7 pg/mL ± 5.8, and 518.4 pg/mL ± 8.8, respectively (Figure 5C,D). IL-6 concentration decreased by 1.05 % (672.8 pg/mL ±13.9) in samples treated with SECO. ED and EL pre-treated conditions decreased the release of IL-6 by approximately 3.74% (690.6 pg/mL ± 5.30) and 26.41% (489.9 pg/mL ± 11.31), respectively. With respect to TNF-α, SECO, ED and EL reduced cytokine release by 3.97% (497.8 pg/mL ± 12.42), 0.5% (515.8 pg/mL ± 7.12) and 29.47% (365.6 pg/mL ± 4.91), respectively. Like GEN above, EL showed significantly greater reduction of inflammatory cytokines when compared to SECO and EL (Tables S4–S6).

## 3. Discussion

The overall health of humans and communities depends on many factors including genetics, various environmental factors, and diet. Increased adherence to the Mediterranean diet was recently correlated with a reduced risk of neurodegenerative diseases, including prodromal Parkinson’s disease [5,42]. In this report, we sought to explore two common classes of polyphenols found in the Mediterranean diet, namely isoflavones and lignans, to investigate the bioavailability and bioactivity of these compounds and their metabolites.

Previously published literature suggests that for polyphenols to be bioactive, they must first be transformed in the colon by the gut microbiota into molecularly unique metabolites [16]. Compounds which are permeable through the gut mucosa enter the bloodstream and circulate in the body. The in vivo permeability may change if these substances are compatible with active transporters, as well as differences based on the individual’s GI environment and diet [43]. A number of factors contribute to the absorption ability of a compound, namely, physicochemical (e.g., pKa, solubility, polarity), physiological (e.g., GI pH, GI blood flow), and dosage form (e.g., tablet, capsule) [44].

To investigate the role of lignans, isoflavones, and PMMs in systemic circulation, we explored gut permeability through in silico (SwissADME) and in vitro (PAMPA) models. Gut PAMPA results largely coincided with those of in silico predictions and showed that the test compounds, with the exception of ED, passively permeate the gut. The presence of O-methyls on SECO and lactone moiety on EL may provide these molecules with additional lipophilicity needed to cross the membrane (Figure 1), whereas ED contains free hydroxyl groups, which may reduce passive permeability. These data are supported by previously published results such as the evaluation of GI absorption of alkaloids from *Coptis* [45]. PAMPA is a useful tool for predicting a compound’s permeability through the human gut, however, it only considers the rate of passive transport [36].

The BBB serves as a protective barrier of the brain, comprised of endothelial cells that create tight junctions, allowing for extremely selective passive permeability [40]. This typically results in higher penetrability by lipophilic compounds through the BBB [46]. Compounds that pass through the BBB may influence the brain state and physiology. We sought to identify if the aforementioned isoflavones, lignans, and PMMs can passively cross the BBB and could be relevant in modulating the pathogenic features of neurodegenerative diseases.

Computational predictive software, SwissADME, identified EL, DAI and EQ as BBB-penetrable molecules. Interestingly, neither GEN nor SECO were considered permeable. Furthermore, our PAMPA in vitro study supported these results, reporting high permeability of EL and EQ. SECO and ED exhibited no apparent permeability. The parent compounds of isoflavones, GEN and DAI, were identified as intermediate permeable compounds. Similar investigative strategies have identified high permeability of compounds derived from natural sources through PAMPA–BBB [47]. While there are many other factors that contribute to BBB permeability, such as plasma concentration, plasma binding, and metabolic modifications by barrier enzymes, PAMPA is a good predictor of passive movement across the BBB [35,46]. The relationship between gut and BBB permeability is further complicated by presystemic metabolism. For instance, previous studies have shown that following gut metabolism, EL predominantly exists as a glucuronide conjugate, certainly affecting its BBB permeability and overall bioavailability [48].

We identified that these PMMs, specifically EL and EQ, passively cross both the gut and BBB barriers. To further identify the potential bioactivities of these molecules, we examined their protective ability against inflammation in microglia. Microglia are the resident macrophages of the brain that work to eliminate debris from the brain [48]. However, continuous activation of microglia can lead to the over-production of inflammatory cytokines, as identified in postmortem brains of Alzheimer’s and Parkinson’s disease patients [49]. One mechanism of microglia activation is the increase in LPS levels in peripheral blood, due to poor diet [50,51]. The continued elevated levels of these pro-inflammatory cytokines, specifically IL-6 and TNF-α, can stimulate the recruitment and activation of other microglia, further inducing the production of reactive oxygen species and nitric oxide species [52]. Nitric oxide species at low doses is critical for maintaining healthy microglia and neuron function, but at high doses can induce necrosis or apoptosis [53]. Phenolic compounds have previously been linked to reduced microglia-induced neuroinflammation [54]. At 20 µM, all tested isoflavones were able to significantly reduce nitric oxide production, and TNF-α and IL-6 concentrations compared to LPS alone. These results are consistent with previous reports of isoflavones [14]. However, GEN clearly showed the greatest anti-inflammatory properties when compared to SECO and EQ. Furthermore, we subjected SECO and its microbial-derived metabolites to neuroprotective assays. Among the lignans tested, only gut-derived metabolite EL was able to significantly reduce IL-6 and TNF-α production. Additionally, EL showed significantly greater reduction in nitric oxide concentrations than its parent metabolite and ED. Recent studies with certain polyphenol metabolites, namely gallic acid derivatives, demonstrated that these metabolites pass across BBB endothelium and provide neuroprotective effects through modulation of the NF-κB pathway [55]. It is clear from these previous studies and the work presented here that the neuroprotective potential of BBB-permeable metabolites is an important area for further investigation to understand the relationship between dietary intake of polyphenols and brain health.

In summary, the fate of microbial metabolites is largely governed by their ability to permeate through biological barriers. After investigating two classes of polyphenols often found in the Mediterranean diet, namely, isoflavones and lignans, our data suggest that their gut microbial metabolites, but not parent compounds, may enter the blood, cross the BBB and provide protection against neuroinflammation. The isoflavone parent compound, DAI, exhibits high gut permeability, but intermediate BBB permeability. DAI’s microbial metabolite, EQ, exhibits high permeability in both the gut and BBB, as well as significantly reducing nitric oxide and the production of pro-inflammatory cytokines in murine microglia. GEN shows permeability through both the gut and BBB and very strong anti-inflammatory effects. However, gut microbial metabolism would likely limit the circulating levels of GEN in the blood. The lignan SECO has extremely limited membrane permeability, but its gut microbial metabolite EL passively penetrates both the gut and BBB. Additionally, EL was most effective out of the lignans tested in inhibiting nitric oxide species and pro-inflammatory cytokines in vitro.

These data, supported by previously published literature, suggest the further investigation of gut-microbial-derived metabolites, specifically EL and EQ, for the treatment of neurodegenerative diseases both in vitro and in vivo. Furthermore, using a system like PAMPA can aid in refining in vitro testing to ultimately inform the design of more efficacious animal studies.

## 4. Materials and Methods

### 4.1. Compounds and Chemicals

Dimethylsulfoxide (DMSO) and lipopolysaccharide (LPS) were purchased from Sigma-Aldrich Chemical Co. (St. Louis, MO, USA). Daidzein (DAI), (±) enterodiol (ED) (Cat# 45198-5MG-F), (±) enterolactone (EL) (Cat# 45199-1MG-F), genistein (GEN) and (±) secoisolariciresinol (SECO) (Cat# 60372-5MG-F) were purchased from Sigma-Aldrich Chemical Co. (St. Louis, MO, USA). *S*-Equol was purchased from Chembest (Shanghai, China). Antipyrine, corticosterone, ketoprofen, ranitidine, theophylline and verapamil were purchased as PAMPA controls from Pion, Inc. (Billerica, MA, USA).

### 4.2. In Silico ADME Predictors

The in silico tool SwissADME was used as a measure to predict BBB and gut permeability. Predicted permeability was assessed for each individual compound and control, as previously explained [56].

### 4.3. Parallel Artificial Membrane Permeability Assay (PAMPA)

Parallel Artificial Membrane Permeability Assay was performed to analyze both blood–brain barrier (BBB) and gut passive permeability. All materials for PAMPA were purchased from Pion, Inc (Billerica, MA, USA). Both the gut and BBB assays were performed according to the manufacturer’s instructions. Briefly, test compounds and standards were prepared at 10 mM concentrations in DMSO. For the gut assay, Prisma HT buffer was adjusted to pHs 5, 6.2, and 7.4 by adding 0.5 M NaOH. In the deepwell plate provided by Pion, 1 mL of each pH-adjusted buffer was added to separate wells. For each pH, 5 µL of the sample was added to the 1 mL of buffer then mixed thoroughly with a pipette, diluting the sample to a final concentration of 50 µM. Next, 200 µl of the diluted sample was added to the donor (bottom) plate from the Pion sandwich assay, 5 µL of GIT lipid matrix was added to the membrane on the bottom of the acceptor plate, and 200 µL of acceptor sink buffer was added in each well of the acceptor plate. The sandwich was assembled, and the plate incubated at room temperature for 4 h undisturbed. In BBB assays, compounds (10 mM) were diluted to final concentrations of 50 µM in Prisma HT Buffer (pH = 7.4). The PAMPA sandwich was created with the donor (bottom), BBB artificial membrane, and brain sink buffer as the acceptor (top). The assay was performed for 1 h at room temperature with shaking (60 µm) using the manufacturer-supplied GIT Box. After incubation, the UV profiles of the donor and acceptor plates were read on the SpectraMax plate reader connected to the PAMPA software. PAMPA software calculated the –log P_e_ values for each compound and standard using the UV profiles of the donor and acceptor plates. Standards were all within 0.25 units of the expected value provided by PAMPA and each sample was averaged with 4 replicates.

### 4.4. Cell Culture Conditions

Murine microglia (BV-2) were generously provided by Dr. Grace Sun of the University of Missouri at Columbia. Cells were maintained in DMEM/F12 (Life Technologies, Gaithersburg, MD, USA) supplemented with 10% (*v*/*v*) sterile filtered and heat inactivated Fetal Bovine Serum in addition to 1% (*v*/*v*) Penicillin/Streptomycin Antibiotic Solution (Life Technologies, Gaithersburg, MD, USA). Cells were incubated at approximately 65% relative humidity at 37 °C under atmospheric oxygen and 5% CO_2_. Cells were passaged using Trypsin-EDTA (Life Technologies, Gaithersburg, MD USA), enumerated, and checked for viability using Trypan blue (Sigma Aldrich, St. Louis, MO, USA) dye exclusion. DMSO (Sigma Aldrich, St. Louis, MO, USA) was used to dissolve compounds and did not exceed 0.1% when incubated with cells.

### 4.5. Cell Viability

The cytotoxicity of each compound was determined in BV-2. Briefly, cells were seeded in 96 white-walled, clear bottom plates at 100,000 cells/mL. Cells were allowed to adhere for 24 h. Compounds were prepared to 10 mM in DMSO, then diluted in serum-free media to yield concentrations of 20 and 10 μM. Cells were treated with prepared concentrations of each compound for 24 h. Cellular viability was determined as a percentage of control (DMSO) by Cell Titer Glo 2.0 (CTG; Promega, Madison, WI, USA), read for luminescence on the SpectraMax M2 plate reader (Molecular Devices, Sunnyvale, CA, USA).

### 4.6. LPS Stimulation of Murine Microglia BV-2 Cells

As previously published, BV-2 cells were seeded at a density of 100,000 cells/mL in 24-well plates [57]. After reaching a confluency of 85%, cells were exposed to either DMSO, SECO (10 μM), ED (10 μM), EL (10 μM), GEN (20, 10 μM), DAI (20, 10 μM), or EQ (20, 10 μM), for 1 h prior to incubation of LPS (1 μg/mL) for 23 h. Media were collected, aliquoted for nitric oxide and cytokine analysis.

### 4.7. Quantification of Nitric Oxide

Nitric oxide was detected in BV-2 culture media following the stimulation of LPS for 24 h from the previously described experiment by way of the Griess Assay (Promega, Madison WI), according to the manufacturer’s protocol.

### 4.8. Measurement of IL-6 and TNF-α

IL-6 and TNF-α were measured from BV-2 media using Enzyme Linked Immunosorbent Assay (ELISA) provided by Biolegend (San Diego, CA, USA) [57].

### 4.9. Statistical Analysis

All data are reported as mean ± standard errors of at least three independent biological samples. The analysis of all cellular data was conducted by ANOVA followed by Dunnett’s test or Tukey’s test for multiple comparisons of group means. The significance of the toxic agent compared to the control group is presented as *p* ≤ 0.001 (###) and *p* ≤ 0.0001 (####). The significance for tests compared to toxic treatment was defined as: *p* ≤ 0.03 (*), *p* ≤ 0.002 (**), *p* ≤ 0.0002 (***) and *p* ≤ 0.0001 (****). GraphPad Prism software 7.0 (GraphPad Software, Inc., San Diego, CA, USA) was used for all statistical analysis calculations and graphical representations.

## Figures and Tables

**Table 1:** In silico SwissADME predictive permeability of parent polyphenols and their microbial metabolites in the gut and blood–brain barrier (BBB).

Compound	Molecular Weight (g/mol)	Gut Absorption	BBB Permeability
SECO	362.42	High	No
ED	302.36	High	No
EL	298.33	High	Yes
GEN	270.24	High	No
DAI	254.24	High	Yes
EQ	242.27	High	Yes
Antipyrine	188.23	High	Yes
Corticosterone	346.46	High	Yes
Ketoprofen	254.28	High	Yes
Ranitidine	314.40	High	No
Theophylline	180.16	High	No
Verapamil	454.60	High	Yes

## Supplementary Materials

The following are available online at https://www.mdpi.com/2218-1989/9/4/78/s1, Table S1: Effect of isoflavones (GEN, DAI, EQ) on NOS production, Table S2: Effect of isoflavones (GEN, DAI, EQ) on TNF-α concentration, Table S3: Effect of isoflavones (GEN, DAI, EQ) on IL-6 concentration, Table S4: Effect of lignans (SECO, ED, EL) on NOS production, Table S5: Effect of lignans (SECO, ED, EL) on TNF-α production, Table S6: Effect of lignans (SECO, ED, EL) on IL-6 production.
