# Supplementary material for: Polyphenol Microbial Metabolites Exhibit Gut and Blood–Brain Barrier Permeability and Protect Murine Microglia against LPS-Induced Inflammation
Source: Metabolites. 2019 Apr 19;9(4):78. doi: 10.3390/metabo9040078 (PMC6523162; doi:10.3390/metabo9040078)
Supplement: Supplementary file 1 [file metabolites-09-00078-s001.pdf]

Supplementary Material

Polyphenol microbial metabolites exhibit gut and blood-brain barrier permeability and protect murine microglia against LPS-induced inflammation

Shelby L. Johnson [1,2], Riley D. Kirk [2], Nicholas A. DaSilva[2], Hang Ma[1,2], Navindra P. Seeram[1,2,*] and Matthew J. Bertin[2,*]

[1] George and Anne Ryan Institute for Neuroscience, University of Rhode Island, Kingston, RI 02881, USA
[2] Department of Biomedical and Pharmaceutical Sciences, College of Pharmacy, University of Rhode Island, Kingston, RI 02881, USA
* Correspondence: nseeram@uri.edu, Tel. 401-874-9367 (N.P.S.); mbertin@uri.edu, Tel.: 401-874-5016 (M.J.B.)

Received: date; Accepted: date; Published: date

Table S1. Effect of isoflavones (GEN, DAI, EQ) on NOS production. One-way ANOVA followed by Tukey's multiple comparison procedure. GEN 20 μM comparisons in bold.

Tukey's multiple comparisons test	Mean Diff.	95.00% CI of diff.	Significant?	Summary	Adjusted P Value
Control vs. LPS (1 μg/mL)	-36.28	-43.18 to -29.38	Yes	****	<0.0001
Control vs. GEN (20 μM)	-10.11	-17.27 to -2.941	Yes	**	0.0011
Control vs. GEN (10 μM)	-21.87	-29.04 to -14.7	Yes	****	<0.0001
Control vs. DAI (20 μM)	-27.05	-34.22 to -19.89	Yes	****	<0.0001
Control vs. DAI (10 μM)	-32.15	-39.31 to -24.98	Yes	****	<0.0001
Control vs. EQ (20 μM)	-27.81	-34.97 to -20.64	Yes	****	<0.0001
Control vs. EQ (10 μM)	-30.06	-37.23 to -22.9	Yes	****	<0.0001
LPS (1 μg/mL) vs. GEN (20 μM)	26.17	18.64 to 33.7	Yes	****	<0.0001
LPS (1 μg/mL) vs. GEN (10 μM)	14.41	6.88 to 21.93	Yes	****	<0.0001
LPS (1 μg/mL) vs. DAI (20 μM)	9.223	1.697 to 16.75	Yes	**	0.0069
LPS (1 μg/mL) vs. DAI (10 μM)	4.129	-3.397 to 11.66	No	ns	0.6667
LPS (1 μg/mL) vs. EQ (20 μM)	8.47	0.9441 to 16	Yes	*	0.0173
LPS (1 μg/mL) vs. EQ (10 μM)	6.216	-1.311 to 13.74	No	ns	0.1766
**GEN (20 μM) vs. GEN (10 μM)**	**-11.76**	**-19.54 to -3.991**	**Yes**	*******	**0.0004**
**GEN (20 μM) vs. DAI (20 μM)**	**-16.95**	**-24.72 to -9.174**	**Yes**	********	**<0.0001**
**GEN (20 μM) vs. DAI (10 μM)**	**-22.04**	**-29.81 to -14.27**	**Yes**	********	**<0.0001**
**GEN (20 μM) vs. EQ (20 μM)**	**-17.7**	**-25.47 to -9.927**	**Yes**	********	**<0.0001**
**GEN (20 μM) vs. EQ (10 μM)**	**-19.95**	**-27.73 to -12.18**	**Yes**	********	**<0.0001**
GEN (10 μM) vs. DAI (20 μM)	-5.184	-12.96 to 2.59	No	ns	0.425
GEN (10 μM) vs. DAI (10 μM)	-10.28	-18.05 to -2.504	Yes	**	0.0027
GEN (10 μM) vs. EQ (20 μM)	-5.936	-13.71 to 1.837	No	ns	0.2577
GEN (10 μM) vs. EQ (10 μM)	-8.191	-15.96 to -0.418	Yes	*	0.0322
DAI (20 μM) vs. DAI (10 μM)	-5.094	-12.87 to 2.679	No	ns	0.4477

DAI (20 µM) vs. EQ (20 µM)	-0.7528	-8.526 to 7.02	No	ns	>0.9999
DAI (20 µM) vs. EQ (10 µM)	-3.008	-10.78 to 4.766	No	ns	0.9217
DAI (10 µM) vs. EQ (20 µM)	4.341	-3.432 to 12.11	No	ns	0.6467
DAI (10 µM) vs. EQ (10 µM)	2.086	-5.687 to 9.859	No	ns	0.9893
EQ (20 µM) vs. EQ (10 µM)	-2.255	-10.03 to 5.518	No	ns	0.9832

Table S2. Effect of isoflavones (GEN, DAI, EQ) on TNF-α concentration. One-way ANOVA followed by Tukey's multiple comparison procedure. GEN 20 µM comparisons in bold.

Tukey's multiple comparisons test	Mean Diff.	95.00% CI of diff.	Significant?	Summary	Adjusted P Value
Control vs. LPS (1 µg/mL)	-572.2	-608.4 to -536	Yes	****	<0.0001
Control vs. EST (20 µM)	-539.1	-581.6 to -496.7	Yes	****	<0.0001
Control vs. EST (10 µM)	-545.6	-588.1 to -503.1	Yes	****	<0.0001
Control vs. GEN (20 µM)	-372.4	-414.9 to -330	Yes	****	<0.0001
Control vs. GEN (10 µM)	-458.4	-500.9 to -415.9	Yes	****	<0.0001
Control vs. DAI (20 µM)	-501.7	-544.1 to -459.2	Yes	****	<0.0001
Control vs. DAI (10 µM)	-514.3	-556.8 to -471.9	Yes	****	<0.0001
Control vs. EQ (20 µM)	-499.5	-541.9 to -457	Yes	****	<0.0001
Control vs. EQ (10 µM)	-515.2	-557.7 to -472.7	Yes	****	<0.0001
LPS (1 µg/mL) vs. EST (20 µM)	33.1	-9.363 to 75.55	No	ns	0.2428
LPS (1 µg/mL) vs. EST (10 µM)	26.62	-15.84 to 69.08	No	ns	0.5312
LPS (1 µg/mL) vs. GEN (20 µM)	199.8	157.3 to 242.2	Yes	****	<0.0001
LPS (1 µg/mL) vs. GEN (10 µM)	113.8	71.38 to 156.3	Yes	****	<0.0001
LPS (1 µg/mL) vs. DAI (20 µM)	70.55	28.09 to 113	Yes	****	<0.0001
LPS (1 µg/mL) vs. DAI (10 µM)	57.91	15.46 to 100.4	Yes	**	0.0019
LPS (1 µg/mL) vs. EQ (20 µM)	72.78	30.32 to 115.2	Yes	****	<0.0001
LPS (1 µg/mL) vs. EQ (10 µM)	57.03	14.57 to 99.49	Yes	**	0.0023
**GEN (20 µM) vs. GEN (10 µM)**	**-85.94**	**-133.8 to -38.04**	**Yes**	****	**<0.0001**
**GEN (20 µM) vs. DAI (20 µM)**	**-129.2**	**-177.1 to -81.34**	**Yes**	****	**<0.0001**
**GEN (20 µM) vs. DAI (10 µM)**	**-141.9**	**-189.8 to -93.97**	**Yes**	****	**<0.0001**
**GEN (20 µM) vs. EQ (20 µM)**	**-127**	**-174.9 to -79.11**	**Yes**	****	**<0.0001**
**GEN (20 µM) vs. EQ (10 µM)**	**-142.8**	**-190.6 to -94.85**	**Yes**	****	**<0.0001**
GEN (10 µM) vs. DAI (20 µM)	-43.29	-91.19 to 4.608	No	ns	0.1051
GEN (10 µM) vs. DAI (10 µM)	-55.92	-103.8 to -8.024	Yes	*	0.0118
GEN (10 µM) vs. EQ (20 µM)	-41.06	-88.96 to 6.837	No	ns	0.1464
GEN (10 µM) vs. EQ (10 µM)	-56.81	-104.7 to -8.906	Yes	*	0.01
DAI (20 µM) vs. DAI (10 µM)	-12.63	-60.53 to 35.27	No	ns	0.9959
DAI (20 µM) vs. EQ (20 µM)	2.229	-45.67 to 50.13	No	ns	>0.9999
DAI (20 µM) vs. EQ (10 µM)	-13.51	-61.41 to 34.39	No	ns	0.9933
DAI (10 µM) vs. EQ (20 µM)	14.86	-33.04 to 62.76	No	ns	0.987
DAI (10 µM) vs. EQ (10 µM)	-0.8819	-48.78 to 47.02	No	ns	>0.9999
EQ (20 µM) vs. EQ (10 µM)	-15.74	-63.64 to 32.16	No	ns	0.9808

Table S3. Effect of isoflavones (GEN, DAI, EQ) on IL-6 concentration. One-way ANOVA followed by Tukey's multiple comparison procedure. GEN 20 µM comparisons in bold.

Tukey's multiple comparisons test	Mean Diff.	95.00% CI of diff.	Significant?	Summary	Adjusted P Value
Control vs. LPS (1 µg/mL)	-103.1	-112.4 to -93.84	Yes	****	<0.0001
Control vs. EST (20 µM)	-83.66	-94.53 to -72.8	Yes	****	<0.0001
Control vs. EST (10 µM)	-89.07	-99.94 to -78.21	Yes	****	<0.0001
Control vs. GEN (20 µM)	-19.72	-30.59 to -8.858	Yes	****	<0.0001
Control vs. GEN (10 µM)	-67.96	-78.82 to -57.09	Yes	****	<0.0001
Control vs. DAI (20 µM)	-82.42	-93.29 to -71.56	Yes	****	<0.0001
Control vs. DAI (10 µM)	-85.84	-96.71 to -74.98	Yes	****	<0.0001
Control vs. EQ (20 µM)	-83.63	-94.49 to -72.76	Yes	****	<0.0001
Control vs. EQ (10 µM)	-92.72	-103.6 to -81.85	Yes	****	<0.0001
LPS (1 µg/mL) vs. EST (20 µM)	19.44	8.575 to 30.31	Yes	****	<0.0001
LPS (1 µg/mL) vs. EST (10 µM)	14.03	3.169 to 24.9	Yes	**	0.0037
LPS (1 µg/mL) vs. GEN (20 µM)	83.38	72.52 to 94.25	Yes	****	<0.0001
LPS (1 µg/mL) vs. GEN (10 µM)	35.15	24.28 to 46.01	Yes	****	<0.0001
LPS (1 µg/mL) vs. DAI (20 µM)	20.68	9.818 to 31.55	Yes	****	<0.0001
LPS (1 µg/mL) vs. DAI (10 µM)	17.26	6.397 to 28.13	Yes	***	0.0002
LPS (1 µg/mL) vs. EQ (20 µM)	19.48	8.614 to 30.34	Yes	****	<0.0001
LPS (1 µg/mL) vs. EQ (10 µM)	10.39	-0.4786 to 21.25	No	ns	0.0709
**GEN (20 µM) vs. GEN (10 µM)**	**-48.23**	**-60.49 to -35.98**	**Yes**	****	**<0.0001**
**GEN (20 µM) vs. DAI (20 µM)**	**-62.7**	**-74.96 to -50.44**	**Yes**	****	**<0.0001**
**GEN (20 µM) vs. DAI (10 µM)**	**-66.12**	**-78.38 to -53.86**	**Yes**	****	**<0.0001**
**GEN (20 µM) vs. EQ (20 µM)**	**-63.9**	**-76.16 to -51.64**	**Yes**	****	**<0.0001**
**GEN (20 µM) vs. EQ (10 µM)**	**-72.99**	**-85.25 to -60.74**	**Yes**	****	**<0.0001**
GEN (10 µM) vs. DAI (20 µM)	-14.46	-26.72 to -2.207	Yes	*	0.0106
GEN (10 µM) vs. DAI (10 µM)	-17.89	-30.14 to -5.628	Yes	***	0.0007
GEN (10 µM) vs. EQ (20 µM)	-15.67	-27.93 to -3.411	Yes	**	0.0042
GEN (10 µM) vs. EQ (10 µM)	-24.76	-37.02 to -12.5	Yes	****	<0.0001
DAI (20 µM) vs. DAI (10 µM)	-3.421	-15.68 to 8.837	No	ns	0.9938
DAI (20 µM) vs. EQ (20 µM)	-1.204	-13.46 to 11.05	No	ns	>0.9999
DAI (20 µM) vs. EQ (10 µM)	-10.3	-22.55 to 1.961	No	ns	0.1647
DAI (10 µM) vs. EQ (20 µM)	2.216	-10.04 to 14.47	No	ns	0.9998
DAI (10 µM) vs. EQ (10 µM)	-6.876	-19.13 to 5.382	No	ns	0.6755
EQ (20 µM) vs. EQ (10 µM)	-9.092	-21.35 to 3.165	No	ns	0.3028

Table S4. Effect of lignans (SECO, ED, EL) on NOS production. One-way ANOVA followed by Tukey's multiple comparison procedure. EL 10 μM comparisons in bold.

Tukey's multiple comparisons test	Mean Diff.	95.00% CI of diff.	Significant?	Summary	Adjusted P Value
Control vs. LPS (1 μg/mL)	-39.03	-42.1 to -35.97	Yes	****	<0.0001
Control vs. SECO (10 μM)	-34.38	-37.44 to -31.32	Yes	****	<0.0001
Control vs. ED (10 μM)	-34.68	-37.74 to -31.62	Yes	****	<0.0001
Control vs. EL (10 μM)	-23.7	-26.76 to -20.64	Yes	****	<0.0001
LPS (1 μg/mL) vs. SECO (10 μM)	4.653	1.591 to 7.715	Yes	**	0.0016
LPS (1 μg/mL) vs. ED (10 μM)	4.354	1.292 to 7.417	Yes	**	0.0031
LPS (1 μg/mL) vs. EL (10 μM)	15.34	12.27 to 18.4	Yes	****	<0.0001
SECO (10 μM) vs. ED (10 μM)	-0.2985	-3.361 to 2.764	No	ns	0.9995
**SECO (10 μM) vs. EL (10 μM)**	**10.68**	**7.62 to 13.75**	**Yes**	****	**<0.0001**
**ED (10 μM) vs. EL (10 μM)**	**10.98**	**7.919 to 14.04**	**Yes**	****	**<0.0001**

Table S5. Effect of lignans (SECO, ED, EL) on TNF-α production. One-way ANOVA followed by Tukey's multiple comparison procedure. EL 10 μM comparisons in bold.

Tukey's multiple comparisons test	Mean Diff.	95.00% CI of diff.	Significant?	Summary	Adjusted P Value
Control vs. LPS (1 μg/mL)	-491.3	-523.1 to -459.4	Yes	****	<0.0001
Control vs. SECO (10 μM)	-470.7	-502.5 to -438.8	Yes	****	<0.0001
Control vs. ED (10 μM)	-488.6	-520.5 to -456.8	Yes	****	<0.0001
Control vs. EL (10 μM)	-338.5	-370.3 to -306.7	Yes	****	<0.0001
LPS (1 μg/mL) vs. SECO (10 μM)	20.62	-11.21 to 52.44	No	ns	0.3557
LPS (1 μg/mL) vs. ED (10 μM)	2.638	-29.18 to 34.46	No	ns	0.9992
LPS (1 μg/mL) vs. EL (10 μM)	152.8	120.9 to 184.6	Yes	****	<0.0001
SECO (10 μM) vs. ED (10 μM)	-17.98	-49.8 to 13.84	No	ns	0.4925
**SECO (10 μM) vs. EL (10 μM)**	**132.1**	**100.3 to 164**	**Yes**	****	**<0.0001**
**ED (10 μM) vs. EL (10 μM)**	**150.1**	**118.3 to 181.9**	**Yes**	****	**<0.0001**

Table S6. Effect of lignans (SECO, ED, EL) on IL-6 production. One-way ANOVA followed by Tukey's multiple comparison procedure. EL 10 μM comparisons in bold.

Tukey's multiple comparisons test	Mean Diff.	95.00% CI of diff.	Significant?	Summary	Adjusted P Value
LPS (1 μg/mL) vs. SECO (10 μM)	-7.108	-44.93 to 30.72	No	ns	0.9553
LPS (1 μg/mL) vs. ED (10 μM)	-24.9	-62.72 to 12.93	No	ns	0.2958
LPS (1 μg/mL) vs. EL (10 μM)	175.8	138 to 213.6	Yes	****	<0.0001
SECO (10 μM) vs. ED (10 μM)	-17.79	-55.61 to 20.03	No	ns	0.5803
**SECO (10 μM) vs. EL (10 μM)**	**182.9**	**145.1 to 220.7**	**Yes**	****	**<0.0001**
**ED (10 μM) vs. EL (10 μM)**	**200.7**	**162.9 to 238.5**	**Yes**	****	**<0.0001**
